# Changes in salivary oxytocin in response to biologically-relevant events in farm animals: method optimization and usefulness as a biomarker

**DOI:** 10.3389/fphys.2024.1370557

**Published:** 2024-03-19

**Authors:** Liza R. Moscovice, Birgit Sobczak, Taru Niittynen, Sonja E. Koski, Ulrike Gimsa

**Affiliations:** ^1^ Psychophysiology Working Group, Research Institute for Farm Animal Biology, Dummerstorf, Germany; ^2^ Organismal and Evolutionary Biology Research Programme, University of Helsinki, Helsinki, Finland

**Keywords:** neuropeptide, parturition, social separation, pigs, horses, non-invasive

## Abstract

Although best known for its established role in mediating parturition and lactation, the highly-conserved neuropeptide hormone oxytocin also mediates a range of social and stress-buffering processes across mammalian species. Measurements of peripheral oxytocin in plasma have long been considered the gold standard, but there is increasing interest in developing methods to detect oxytocin non-invasively in saliva. Here we present an analytical and biological validation of a novel method to measure salivary oxytocin (sOXT) in an under-studied research group: farm animals. Given their similarities with humans in physiology and brain, methods that can identify valued social contexts and social relationships for farm animals and investigate their function have implications for clinical research as well as for animal welfare science. However, current methods to measure sOXT vary greatly in terms of sample collection, pre-measurement processing and measurement and more rigorous standardization and validation of methods is critical to determine the utility of sOXT as a biomarker of salient social events and related emotions. We optimized a method for extracting sOXT in pigs and horses and measured sOXT in extracted samples using a commercially available enzyme-immunoassay. Extracted samples were within acceptable ranges for precision (CVs < 15.2%), parallelism and recovery (94%–99%) in both species. Salivary oxytocin increased in samples collected during birth in pigs (Friedmans, *p* = 0.02) and horses (Wilcoxon, *p* = 0.02). Salivary oxytocin tended to decrease in sows after a 90-min separation from their piglets (Wilcoxon, *p* = 0.08). We conclude that sOXT can be reliably linked to physiological events that are mediated by the oxytocinergic system in farm animals, but that more research is needed to determine whether sOXT is a reliable trait marker for more general oxytocin system activation in response to salient social events. Future research should characterize how individual attributes and salivary parameters influence sOXT measurement and should emphasize reporting of analytical and biological validations to increase acceptance of non-invasive methods.

## 1 Introduction

One of the most ancient and highly-conserved hypothalamic neuropeptides is found across invertebrate and vertebrate taxa, and in mammals is expressed as oxytocin (OXT). The hypothalamus mediates the release of OXT centrally via neural projections to brain regions associated with social behavior (reviewed in Caldwell, 2017) and reward- and fear-processing (reviewed in Marsh et al., 2020). In addition to its actions as a neurotransmitter, OXT can also be released directly into the bloodstream via the hypothalamo-neurohypophyseal axis, where it acts as a neurohormone (reviewed in Donaldson and Young, 2008). Historically, oxytocin has been associated with its endocrine functions in facilitating female reproduction and lactation (reviewed in Borrow and Cameron, 2012; Mota-Rojas et al., 2023). However, recent research has highlighted important roles for oxytocin centrally in mediating social interactions with valued partners (reviewed in Donaldson and Young, 2008), and providing inhibitory feedback to the hypothalamic-pituitary-adrenal (HPA) axis to mediate stress responses (Engelmann et al., 2004). There is also neuroanatomical (Ross et al., 2009) and experimental evidence that oxytocin can be released in a coordinated manner centrally and peripherally in response to salient social behavior (Nyuyki et al., 2011) and stressors (Wotjak et al., 1998). As a result, oxytocin is receiving increased attention in animal welfare science as a potential biomarker of positive emotions in domesticated animals (Rault et al., 2017). Validating methods to measure peripheral oxytocin in a wider range of species and contexts is important to determine its utility and also its limitations as an indicator of emotional responses more generally.

Here we present a novel method to measure peripheral oxytocin non-invasively in an under-studied research group: farm animals. Due to their evolutionary history as herd animals (Driscoll et al., 2009), farm animals are highly social species who can discriminate among individuals (Nawroth et al., 2019), are sensitive to each other’s emotions (Baciadonna et al., 2018), and show evidence for prosocial behavior (Cordoni et al., 2023; Moscovice et al., 2023). Given their similarities with humans in physiology and brain (Lunney et al., 2021), research that can identify preferred social partners and social contexts for farm animals has potential clinical implications and is also important for promoting positive welfare in production settings (Lee et al., 2022). Measurements of peripheral oxytocin in plasma have long been considered the gold standard (McCullough et al., 2013). However, the short half-life of approximately 120 s for oxytocin in plasma (Leng and Sabatier, 2016), along with the invasive nature of sample collection, which can interfere with social behavior and influence emotional states, makes plasma sub-optimal for investigating potential links between oxytocin and emotional responses to social stimuli. Saliva presents a promising alternative matrix to plasma for measuring oxytocin non-invasively. There is evidence that oxytocin has a longer half-life in saliva than in plasma (Huffmeijer et al., 2012), and saliva samples can be collected voluntarily from animals in their home environments by a range of personnel without requiring specialized training or restraint of subjects (reviewed in Cerón et al., 2022). This facilitates repeated sampling over shorter-time intervals while minimizing disruptions to behavior and emotional states. In addition, oxytocin in blood can bind to proteins which can interfere with measurement, and this is less likely to occur in saliva, thus improving measurement accuracy (Leng and Sabatier, 2016).

Research measuring salivary oxytocin in domesticated animals is increasing (see Table 1), with some initially promising results. Increases in salivary oxytocin have been related to parturition in sows (López-Arjona et al., 2020a) and sexual behavior in boars (López-Arjona et al., 2020b). There is also some evidence that changes in salivary oxytocin may reflect affiliative social interactions with humans (e.g., farm animals: Lürzel et al., 2020; dogs: Ogi et al., 2020; horses; Niittynen et al., 2022) and mother-offspring interactions (dogs: MacLean et al., 2018). However, there are currently no widely-accepted guidelines for sample collection, processing and measurement of salivary oxytocin, leading to a great degree of variation in methods and results across labs, even when measuring salivary oxytocin in the same species (e.g., pig saliva: López-Arjona et al., 2020a; Lürzel et al., 2020; Moscovice et al., 2022). Greater reporting of analytical as well as biological validations of methods for measuring oxytocin are needed, to promote quality assurance and to better determine whether salivary oxytocin is a robust and reliable biomarker of specific social contexts and related emotions in farm animals.

**TABLE 1:** Comparison of methods and results from publications measuring salivary oxytocin in domesticated animals.

Species	Saliva collection materials	Pre-measurement extraction?	Measurement	Stimulus	Time frame for measuring changes in sOXT	Changes in sOXT^a^	Citation^b^
Cattle	Sarstedt Salivette® swabs (cows); cotton swabs (calves)	Yes and No (compared methods)	In-house assay using ALphaLISA technology	Parturition (cows), weaning (calves)	Several days post-stimulus	↑ During parturition;	López-Arjona et al. (2021a)
						↓ After weaning	
Dogs	Salimetrics SalivaBio® children’s swab and Sarstedt Salivette®	No	Oxytocin ELISA kits from arbor assays and Cayman Chemical (comparison)	Separation and reunion plus nursing	15 min post- stimulus	↑ Following reunion	MacLean et al. (2018)
Dogs	Sarstedt Salivette® swabs	No	Cayman Chemical oxytocin ELISA kit	Petting by owner	15 min post-stimulus	↑ After stroking	Ogi et al. (2020)
Dogs	Sarstedt Salivette® swabs	No	In-house assay using ALphaLISA technology and Cayman Chemical oxytocin ELISA kit (comparison)	Petting by owner	10 and 15 min post-stimulus	↑ After stroking, but only in dogs who accepted swabs and seemed relaxed during stroking (based on owners’ report)	López-Arjona et al. (2021b)
Horses	Salimetrics Salivabio® children’s swabs	Yes	Cayman Chemical oxytocin ELISA kit	Short training with human	15 min post-stimulus	↑ With more positive social interactions;	Niittynen et al. (2022)
						↓ With signs of discomfort	
Pigs	Sarstedt Salivette® swabs	No	In-house assay using ALphaLISA technology	Lactation	Several days post-stimulus	↑ On first lactation day	López-Arjona et al. (2020a)
Pigs	Sarstedt Salivette® swabs	No	In-house assay using ALphaLISA technology	Sexual behavior and ejaculation (males)	Immediately after and 2 h post-stimulus	↑ During ejaculation	López-Arjona et al. (2020b)
Pigs	Sarstedt Salivette® swabs	No	In-house assay using ALphaLISA technology	Transport to and lairage at slaughter house	Time of arrival and 4 h post-arrival	↓ At 4 h post-arrival	López-Arjona et al. (2020c)
Pigs	Salimetrics Salivabio® infant swabs	Yes	Cayman Chemical oxytocin ELISA kit	Parturition (sows); weaning, group play, separation and reunion (piglets)	1 day post-stimulus (parturition) and 15–60 min post-stimulus (weaning, play, separation and reunion)	↑ During parturition;	Moscovice et al. (2022)
						↔ After weaning, group play and reunions	
Pigs	Cotton buds	No	CUSABIO ELISA kit	Parturition, lactation and reunion with offspring after separation	Several days before (parturition and lactation); 15 min post-stimulus (reunions)	↔ Parturition;	Hall et al. (2021)
						↓ Lactation;	
						↔ Following reunions	
Pigs, cattle and goats	Salimetrics Salivabio® children’s swabs	Yes (pigs and cattle), No (goats)	Cayman Chemical oxytocin ELISA kit	Social contact with familiar or unfamiliar human	15 min post-stimulus	↔ With familiar vs. unfamiliar human;	Lürzel et al. (2020)
						↑ With more positive social interactions in pigs and cattle, but not goats	

^a^ Arrows indicate direction of changes in salivary oxytocin in response to the stimulus (↑ = increase, ↓ = decrease, ↔ = no change).

^b^ For full citations refer to the reference list.

Our method is based on previous approaches to measure salivary oxytocin in pigs by our own (Moscovice et al., 2022) and other labs (Lürzel et al., 2020), and was further optimized for pigs following recommendations by Gnanadesikan and colleagues (Gnanadesikan et al. (2021, 2022). We also show that our optimized method works well for measuring salivary oxytocin in horses. In accordance with recommended guidelines when using commercially available enzyme immunoassays (Andreasson et al., 2015), we performed a partial validation of salivary oxytocin in extracted samples from both species for repeatability or precision (the closeness of agreement between independent test results), parallelism (to determine if the binding characteristics of the endogenous analyte to the kit antibodies are the same as for the kit calibrator) and recovery (to determine if the concentration-response relationship is similar in the samples and in the calibration curve). In addition to these analytical validations, we also performed several biological validations. Given the known role of peripheral oxytocin in inducing smooth muscle contractions during parturition (Borrow and Cameron, 2012), we predicted that sOXT would increase during the birth process in sows and mares. Given the important role of the oxytocinergic system in mediating mother-offspring bonds (Mota-Rojas et al., 2023), we additionally predicted that sOXT would decrease in lactating sows temporarily separated from their offspring.

## 2 Materials and methods

### 2.1 Animals

We collected samples from *n* = 14 primiparous German Landrace sows. All sows were born and raised in the experimental pig facility (EAS) of the FBN. The research procedures for sows were part of a larger study that was approved under the German Animal Welfare Act (German Animal Protection Law, §8 TierSchG) by the Committee for Animal Use and Care of the Agricultural Department of Mecklenburg-Western Pomerania, Germany (permit LALLF 7221.3-1-029/21-1). Sows were kept either in conventional (*n* = 8) or enriched (*n* = 6) farrowing pens. Under conventional conditions, sows were housed in 6 sq. meter loose farrowing pens containing a wood chew toy for enrichment and a heated, covered lying area (90 × 90 cm) for piglets. Sows were fed twice daily with a pregnancy feed (Sow Structure 7.0, Trede & von Pein GmbH, Itzehoe, Germany) until 6 days prior to birth, at which point they were switched to a lactation feed (Sow Provital, Trede & von Pein GmbH, Itzehoe, Germany). From 3 days prior to parturition until 4 days post-birth, a guard rail was employed to reduce piglet crushing (Marchant et al., 2000), which limited the sows available area to approximately 200 × 90 cm. Piglets were offered a prestarter feed (Hakra-Immuno-G, Una Hakra, Hamburg, Germany) from the 2nd week of life and were weaned at 28 days of age. In enriched farrowing pens, sows had 8.4 sq. meter pens with an additional 5 sq. meter outdoor run and were given straw bedding daily. Sows were fed a high-fiber, energy-supplemented organic lactation feed (CeraGreen, Mecklenburg-Vorpommern, Germany) several times a day. Piglets were weaned at 42 days of age. As part of the standard husbandry practices at the EAS, births are synchronized among sows, and labor was therefore induced by administering 2 mL synthetic prostaglandin (Cloprostenol, PGF Veyx^®^, Provet AG, Lyssach, Switzerland) on the 114th gestation day (1 day prior to the scheduled parturition). Additionally, *n* = 8 sows had a delayed onset of parturition and therefore received 1 mL of an oxytocin analogue (Carbetocin, LongActon^®^, Vetoquinol AG, Bern, Switzerland), on their 115th gestation day to induce contractions. We planned our sample collection to minimize any potential interference from these treatments on sOXT measurement (see Section 2.2).

We also collected samples from *n* = 7 finnhorses from the same farm (Kylämäki farm, southern Finland). All horses were handled regularly by humans. Pregnant mares were kept as a group in an open shed, and hay and water were provided *ad libitum*. When the estimated time of birth was near, or if mares were showing signs of upcoming birth (wax plugs, starting of milk production), they were moved to individual stalls with thick straw bedding and monitored throughout the night. If mares did not give birth, they were returned to the open shed in the morning.

### 2.2 Saliva sampling procedure

Two of the authors (B.S. in pigs and T.N. in horses) used SalivaBio^®^ Childrens Swabs (Salimetrics, California, USA), taped to wooden dowels or plastic rods, to collect saliva samples from sows and mares. Animals were habituated to the procedure, and all animals gave samples voluntarily. We ensured that animals did not eat or drink for 10 min prior to sampling. To obtain samples, we placed the swab in the subjects’ mouth, attempting to sample between the gum and cheek, or under the tongue, where passive drool collects, for up to 2 min. Saturated swabs were inspected and discarded if there were any signs of blood. Swabs were placed in polypropylene tubes on ice. For sows, swabs were centrifuged within 30 min of collection at 4°C (15 min, 2,000 × g) and the eluent was stored at −70°C until hormone analyses. For horses, swabs were moved to a −24°C freezer within an hour of collection and later stored at −80°C until transport to the FBN, which occurred within 2 months of collection. Once at the FBN, swabs were thawed and centrifuged at 4°C (15 min, 2,000 × g) and the eluent was stored at −70°C until analysis. Whenever volumes were sufficient, we stored multiple aliquots of samples for validation steps.

During parturition, *n* = 10 sows and *n* = 7 mares were monitored remotely using video cameras and pens or stalls were only entered to collect saliva samples, unless additional assistance was needed during the birth process. For horses, the birth sample was taken when the amniotic fluid had broken, and contractions had begun. This is the stage in which plasma oxytocin levels are highest in horses (Haluska and Currie, 1988; Vivrette et al., 2000). For sows, we initiated sample collection between 2 and 4 h after the first piglet expulsion, based on other studies indicating peaks in plasma OXT concentrations during this period (Castrén et al., 1993). The timing of sample collection at least 2 hours after the onset of birth also minimized any potential effects of the synthetic oxytocin administration on sOXT measurements, since Carbetocin has an expected half-life of 41 min in sows (Ward et al., 2019). We collected three saliva samples at 5-minute intervals during the birth process, to additionally test for any evidence of spikes in sOXT during birth in sows, similar to evidence for spikes in plasma oxytocin during birth in humans (reviewed in Uvnäs-Moberg et al., 2019). We compared birth samples with additional samples taken outside of the birth process. For horses, we collected between one to three samples at different time points from one to 2 days prior to parturition, when horses were already showing the first signs of labor, including wax plugs and initiation of milk production. For sows, we collected one sample on the day following birth (the first lactation day), between 9:00 and 11:00.

We additionally measured the sOXT responses of *n* = 10 sows to a relevant social context involving separations from their piglets. Six of these sows had also been sampled during their parturition, and four additional sows were not sampled during parturition. Saliva samples were collected from sows during a 90-minute separation from their piglets, which occurred 1 day prior to weaning (lactation day 27 for sows in conventional housing or lactation day 41 for sows in enriched housing). During this time plasma samples were collected from the piglets for a different project. We collected two saliva samples from sows between 7:30 and 11:00. The first sample was taken 10–30 min before the separation from their piglets and the second sample was taken 90 min following the separation, and before sows were reunited with their piglets.

### 2.3 Oxytocin extraction and measurement

#### 2.3.1 Optimization of the extraction procedure

Following recommendations by Gnanadesikan et al. (2021), Gnanadesikan et al. (2022), we optimized our reversed phase solid phase extraction (SPE) procedure with the goals to identify the optimal wash concentration to remove potential contaminants without losing oxytocin, and to identify the optimal elution concentration to elute the majority of OXT in saliva samples. We used as a basis our previously validated SPE procedure for measurement of sOXT in pigs (Moscovice et al., 2022). We extracted samples using sequential elution steps with different concentrations of the eluent acetonitrile (ACN), from 10% to 80%. As samples, we used duplicates of pure OXT standard containing the complete nonapeptide (Cayman Chemicals, MI, USA) at a concentration of 750 pg/mL, and a pooled pig saliva sample with a measured concentration of 220 pg/mL using our previous extraction method. We spiked the pooled sample with an additional 50 pg/mL OXT standard, to increase the total concentration of OXT in the sample. We included the spiked, pooled saliva sample to ensure that the wash and elution steps were also optimized to measure OXT in the matrix of interest. We combined 0.25 mL of each sample with equal parts TFA 0.1%. We used 1 mL, 30 mg sorbent Oasis PRiME^®^ HLB cartridges (Waters Corporation, MA, USA). These cartridges utilize a reversed-phase hydrophilic-lipophilic balance (HLB) chemistry, and a positive pressure manifold. Cartridges were primed with 1 mL 99% ACN/TFA 0.1%, followed by 1 mL TFA 0.1% in water. We then loaded duplicates of each sample (OXT standard or spiked saliva) onto different cartridges. Duplicates were washed with 1 mL or 3 mL TFA 0.1% and sequentially eluted with 1 mL or 3 mL of elution buffer of increasing ACN content. The flow-through from each step in the wash and elution process was collected in glass tubes and evaporated using a vacuum concentrator (SpeedVac^®^ SC210, Thermo Fisher Scientific, MA, USA). Samples were stored in capped glass tubes at −70°C until measurement.

#### 2.3.2 Sample extraction

Before running an assay, samples were thawed on ice and centrifuged at 4°C (5 min, 2,000 × g). We mixed 0.25 mL saliva with equal parts 0.1% trifluoroacetic acid (TFA) in water, vortexed, and centrifuged the samples a second time at 4°C (15 min, 17,000 × g). During preliminary tests, we found that this second centrifugation step in TFA helped to separate out additional particles that may have interfered with oxytocin measurement. We loaded the supernatant on 1 mL, 30 mg sorbent, Oasis PRiME^®^ HLB cartridges (Waters Corporation, MA, USA). Cartridges were equilibrated with 1 mL 99% ACN/TFA 0.1%, followed by 1 mL 0.1% TFA in water. Samples were washed with 1 mL 10% ACN/TFA 0.1% and eluted with 1 mL 60% ACN/TFA 0.1%. Eluents were evaporated using a vacuum concentrator (SpeedVac^®^ SC210, Thermo Fisher Scientific, MA, USA), and stored in capped glass tubes at −70°C until measurement.

#### 2.3.3 Sample measurement

We measured oxytocin with a commercially available enzyme immuno-assay (EIA) from Cayman Chemical (MI, USA, kit no. Cay500440). The assay relies on competitive binding between oxytocin in the samples and an oxytocin-acetylcholinesterase (AChE) conjugate, which compete for binding to an oxytocin polyclonal antiserum captured on a mouse monoclonal anti-rabbit IgG coated-plate. The published mean IC_50_ is 80 pg/mL with a lower limit of detection of 18 pg/mL and a sensitivity of 5.9 pg/mL. The assay has been validated by the manufacturer for the measurement of salivary and plasma OXT in humans. In addition, several studies have found the Cayman assay to be more reliable than others for measurement of oxytocin in other matrices and species (e.g., dogs: MacLean et al., 2018; mice: Gnanadesikan et al., 2021; humans: Gan et al., 2023). Prior to measurement, samples were thawed on ice, combined with 0.25 mL assay buffer (no dilution for extracted samples; 1:2 dilution for unextracted samples), and measured in duplicate, following the kit instructions. Assays were read at 450 nm using a SPECTROstar Nano^®^ calibrated microtiter plate reader (BMG Labtech, Ortenberg, Germany), and calculation of sOXT concentrations was performed using the SPECTROstar Nano^®^ software with a four-parameter logistic fit.

### 2.4 Analytical validations on unextracted and extracted samples

There continues to be debate over the need for extractions of samples in any matrix prior to oxytocin measurement. Proponents argue that extractions help to remove potential sources of interference, such as larger, more abundant proteins, and to concentrate samples when necessary (McCullough et al., 2013; Bienboire-Frosini et al., 2017), while others argue that the extraction process itself may induce sources of error (reviewed in Tabak et al., 2023). We initially checked for parallelism and precision in measurements of unextracted samples in both species, to determine whether an extraction step was necessary. When results from initial validation steps with unextracted samples were satisfactory for a given species, we then completed the analytical validations on unextracted samples for that species. We additionally performed validations on extracted samples for both species. For a subset of samples that had sufficient volumes, we divided these samples into duplicates and measured each duplicate with and without an extraction step, to assess the impact of our extraction method on the measurement of salivary oxytocin.

For precision on unextracted samples, a pooled saliva sample was divided into 7–8 duplicates of 0.25 mL. Each duplicate was combined with 0.25 mL assay buffer (1:2 dilution), vortexed, centrifuged for 1 min (17,000 × g, 4°C) and measured in duplicate on the same plate, following kit instructions. For precision on extracted samples, we created high- and low- concentration pools for each species. To make high concentration pools, we combined reserve aliquots of saliva samples that had previously been extracted and measured as having sOXT concentrations ≥ 140 pg/mL. For low concentration pools, we combined reserve aliquots of saliva samples that had previously been extracted and measured as having sOXT concentrations ≤ 70 pg/mL. Between 7 and 8 duplicates of 0.25 mL each were extracted (see Section 2.3.2), evaporated and frozen at −70°C until measurement. We measured duplicates of pools across multiple plates to calculate inter-assay CVs, and measured duplicates of pools multiple times on the same plate to calculate intra-assay CVs.

For parallelism on unextracted samples, pooled saliva was diluted from 1:2 to 1:32 in assay buffer, vortexed and plated in duplicate. For parallelism on extracted samples, we extracted four duplicates of 0.25 mL each from a pooled saliva sample (see Section 2.3.2). Extracted samples were evaporated and frozen at −70°C. On the day of measurement, samples were thawed and each duplicate was resuspended in 0.2 mL assay buffer, to concentrate samples. Samples were vortexed and centrifuged for 1 min (17,000 × g, 4°C) and the supernatant from all samples was combined into one tube. We then made serial dilutions of the pool from 1:2 to 1:32 in assay buffer, vortexed samples and plated them in duplicate.

We performed recovery of oxytocin in between 2 and 3 different pools from each species. For recovery of unextracted samples, we spiked 225 µL of each pool with 25 µL OXT standard at three different concentrations spanning the linear range (750 pg/mL, 375 pg/mL, 187.5 pg/mL). We added 0.25 mL assay buffer (1:2 dilution) and plated the samples following kit protocols. For recovery of extracted samples, 225 µL duplicates of each pool were spiked with 25 µL OXT standard at three different concentrations spanning the linear range (750 pg/mL, 375 pg/mL, 187.5 pg/mL). Pools were then extracted following the protocol (see Section 2.3.2), reconstituted in 0.25 mL assay buffer (no dilution) and measured following kit instructions.

### 2.5 Statistical analyses

To test for parallelism, we estimated and compared the slopes of the fitted lines between serial dilutions of samples and the kit standard curve, using the “lstrends” and “pairs” functions in the package “lsmeans” (Lenth et al., 2019). To test if sOXT concentrations in extracted and unextracted samples were correlated, we calculated repeated measures correlations (r_rm_), which take into account multiple samples per subject, using the package “rmcorr” (Bakdash and Marusich, 2017). To compare changes in salivary oxytocin in different biological contexts, we used Friedmans tests and Wilcoxon post-hoc comparisons with Bonferroni corrections in the package “rstatix” (Kassambara, 2023). Results of laboratory validations are presented as mean ± SD. Results of biological validations are presented as Medians and 25%–75% inter-quartile ranges (IQRs), consistent with the use of non-parametric tests for the analyses.

## 3 Results

### 3.1 Optimized extraction for measurement of oxytocin in pig saliva

**FIGURE 1:**
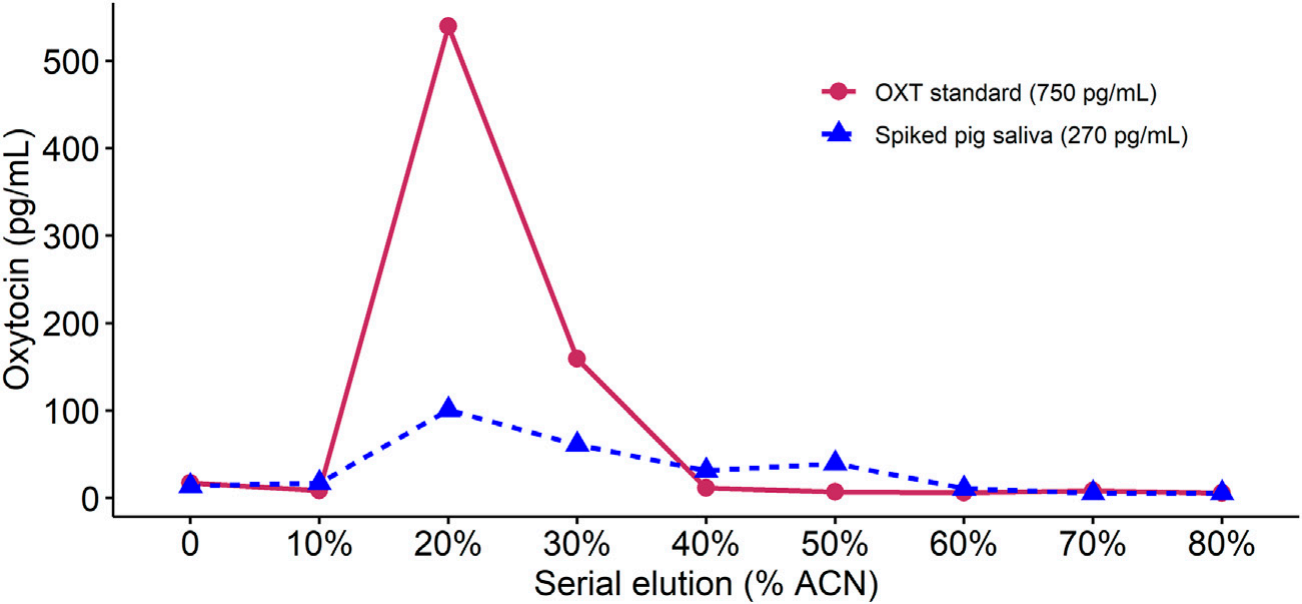
Comparison of oxytocin concentrations detected in an extracted sample of oxytocin standard and an extracted sample containing pig saliva spiked with oxytocin standard. For each sample, we performed sequential 1 mL elutions with increasing acetonitrile (ACN) content (shown on the x-axis). In both cases, no oxytocin was detected at elutions below 20% ACN and the majority of oxytocin eluted at between 20% and 30% ACN concentrations.

When using 1 mL volumes of elution solution with increasing concentrations of ACN, in both the OXT standard and the spiked sOXT sample, negligible concentrations of OXT eluted at 10% ACN, and the majority of OXT eluted at concentrations of between 20% and 30% ACN (Figure 1). However, oxytocin continued to be detected in the spiked saliva sample up to concentrations of 60% ACN, suggesting that this higher ACN concentration may help to capture the majority of OXT in matrix (see Figure 1). We concluded that we could safely wash samples with 1 mL of 10% ACN to target any interfering molecules that may not be removed with weaker wash solutions, but without risking loss of oxytocin. We also confirmed that eluting with 1 mL of 60% ACN would be sufficient to elute the majority of oxytocin in saliva. When increasing the volumes of elution solutions to 3 mL, this resulted in detection of OXT in flow-through in both samples after 3 mL of 10% ACN solution, and an over-estimation of the concentration of the OXT standard after 3 mL of 60% ACN solution (recovery = 126%). We concluded that 1 mL volumes of 10% ACN wash solution and 60% ACN elution solution produced more accurate results.

### 3.2 Analytical validations

#### 3.2.1 Oxytocin can be reliably measured in unextracted saliva from horses, but not pigs

**FIGURE 2:**
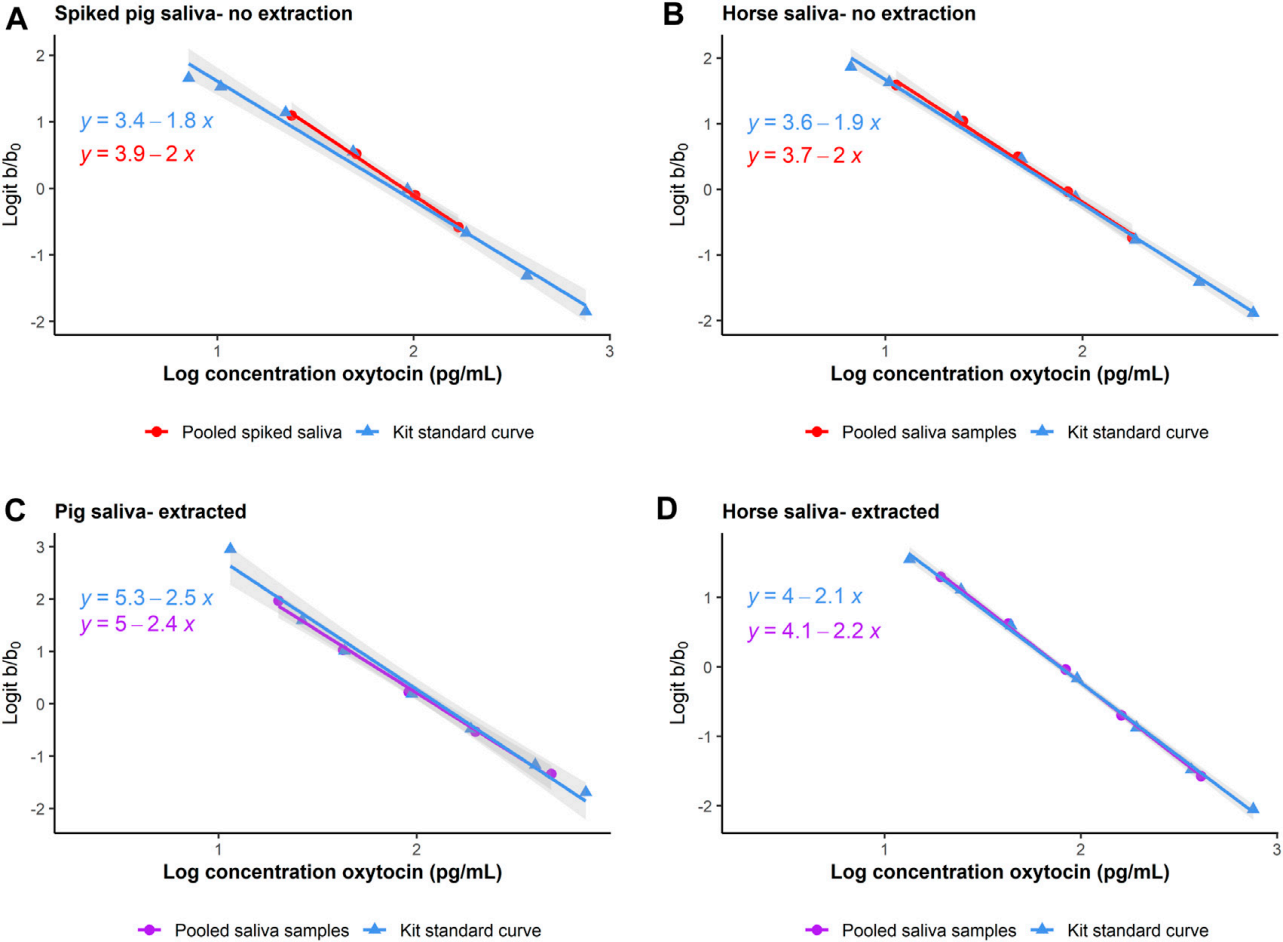
Comparison of parallelism in dilutions of pooled unextracted (**(A, B)**, in red circles) and extracted (**(C, D)**, in purple circles) saliva from pigs and horses, plotted together with the kit standard curve (in blue triangles). In pigs, unextracted saliva was spiked with OXT standard to come within the linear range of the assay. Parallelism is assessed by plotting the log_10_ expected concentrations of samples (x-axis) as a function of the logit of the proportion binding (optical density (b)/maximum binding optical density (b_0_), y-axis). The log10 and logit transformations allow the normally sigmoidal shape of the standard curve to be presented linearly for ease of comparison. Regression lines indicate linear models predicting the expected sample concentrations as a function of binding.

Initial assessments of precision for unextracted pooled pig saliva samples were satisfactory (67.78 (±6.38) pg/mL, CV = 9.41%). In the first attempt at parallelism on unextracted pig saliva, all dilutions were below the limits of detection. In the second attempt, we spiked the pooled pig saliva with 93.75 pg OXT standard to ensure that samples came within the linear range. Dilution linearity on unextracted spiked pig saliva was satisfactory (*t*-test, df = 8, t ratio = −0.832, *p* = 0.429, see Figure 2A). Since we were unable to validate sOXT measurements in unextracted pig samples without spiking, we decided that a reversed phase SPE was necessary prior to measurement of sOXT in pigs.

Precision on unextracted pooled horse samples was satisfactory (127.37 (±7.31) pg/mL, CV = 5.7%). Dilutions of unextracted horse saliva measured within the linear range without spiking, and parallelism was also satisfactory (*t*-test, df = 9, t ratio = −0.581, *p* = 0.575, see Figure 2B). Based on the initially promising results with unextracted horse samples, we also checked and confirmed that recovery on unextracted horse samples was satisfactory (recovery = 105.26 ± 12.13%, *n* = 9 samples).

#### 3.2.2 Oxytocin can be reliably measured in extracted saliva from pigs and horses

Intra-assay CVs for extracted high and low-concentration sOXT pools in pigs were 9.48% (151.87 (±14.40) pg/mL, *n* = 8) and 5.17% (67.01 (±3.47) pg/mL, *n* = 8) respectively. Inter-assay CVs for extracted high- and low-concentration sOXT pools were 13.11% (153.18 ± 20.08 pg/mL, *n* = 8) and 15.20% (61.03 ± 9.27 pg/mL, *n* = 7) respectively. There were no differences in the slopes between serial dilutions of extracted saliva and serial dilutions of the kit OXT standard for pigs (*t*-test, df = 8, t ratio = 0.639, *p* = 0.541, see Figure 2C). Recovery of extracted sOXT in pigs was 94.0% ± 10.2% (*n* = 6 samples).

For horses, intra-assay CVs for extracted high- and low-concentration sOXT pools were 5.5% (160.92 ± 8.87 pg/mL, *n* = 8) and 7.9% (81.85 ± 6.45 pg/mL, *n* = 7) respectively. There were not enough separate test plates containing horse samples to measure inter-assay CVs for horses. There were no differences in the slopes between serial dilutions of extracted horse saliva and serial dilutions of the kit OXT standard (*t*-test, df = 8, t ratio = −1.066, *p* = 0.317, see Figure 2D). Recovery of extracted sOXT in horses was 99.75% ± 11.86% (*n* = 9 samples). In both species, results were within acceptable ranges (e.g., CVs < 20%, recoveries between 80% and 120%, Andreasson et al., 2015).

**FIGURE 3:**
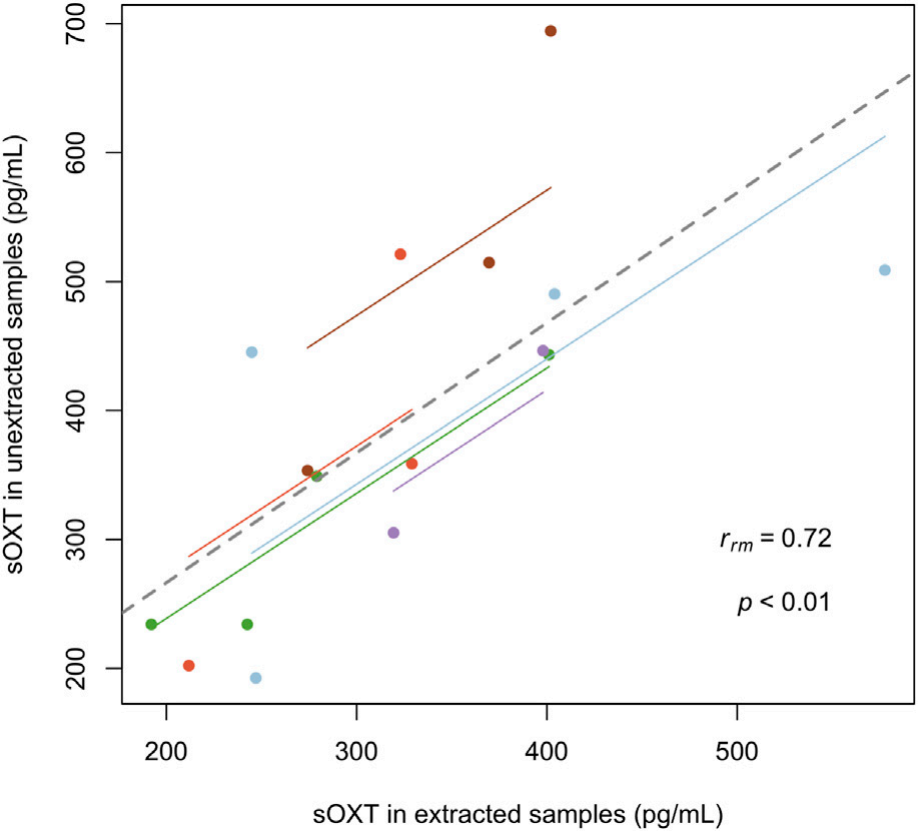
Comparison of salivary oxytocin concentrations in between 2 and 4 samples from *n* = 5 mares (with subjects indicated in different colors). Each point represents one sample that has been measured in duplicate, with and without extraction (r_rm_ = 0.72, CI = 0.26–0.92, df = 10, *p* < 0.01). Regression lines per mare (thin, colored) and overall (thick, dotted) for the repeated measures correlation are indicated.

Since we were able to validate sOXT measurements in horse samples with and without extraction, we also wanted to compare sOXT concentrations in duplicate horse samples run with and without the extraction step. Since many of the horse birth samples did not have sufficient volumes of saliva for duplicate measurements, we used duplicates of n = 16 samples from n = 5 mares (n = 2-4 samples per mare) that were collected at different time points from two days before the birth, to the day of the birth, but not always during the birth process itself. Concentrations of sOXT in mares were strongly correlated in duplicates of samples measured with or without extraction (r_rm_ = 0.72, CI = 0.26-0.92, df = 10, p = 0.008, see Figure 3).

### 3.3 Biological validations

#### 3.3.1 Salivary oxytocin increases in sows and mares during parturition

**FIGURE 4:**
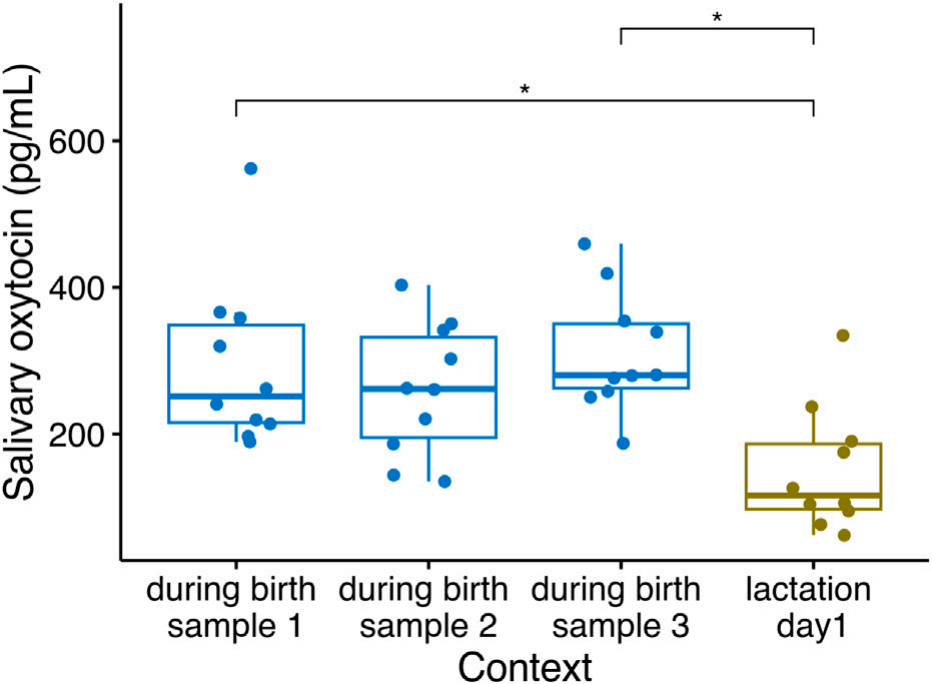
Salivary oxytocin concentrations in *n* = 10 sows sampled at five-minute intervals during parturition and after their parturition had ended, on their first lactation day (Friedmans, *n* = 10, χ^2^(3) = 10.3, *p* = 0.016). Boxplots indicate medians and 25% and 75% interquartile ranges. Error bars represent minimum and maximum values (excluding outliers). *p < 0.05.

Changes in sOXT concentrations have been detected in sows sampled on different days either before parturition or during lactation (e.g., López-Arjona et al., 2020a; Hall et al., 2021), but more precise tracking of peripheral oxytocin fluctuations during the birth process itself have so far only been measured in plasma using catheters (e.g., Castrén et al., 1993). We therefore collected multiple saliva samples at five-minute intervals during the birth process in sows, to characterize the short-term changes in sOXT during birth. We compared these samples with a post-birth sample collected on the first full lactation day following the birth. Salivary OXT concentrations in sows differed across the four sampling time points-at five-minute intervals during the birth and one-day following the birth (Friedmans, *n* = 10, χ^2^(3) = 10.3, *p* = 0.016). Post-hoc tests indicated no differences in sOXT concentrations in the three samples collected during parturition (Median (25%–75% IQR) = 269 (220–349) pg/mL, p adj. > 0.193, Figure 4). However, birth samples at two time points differed from the post-birth sample taken on the first lactation day (Median (IQR) = 116 (97.5–187 pg/mL, p adj. < 0.035, see Figure 4), and birth samples at the third time point tended to differ from the post-birth samples (p adj. = 0.059).

**FIGURE 5:**
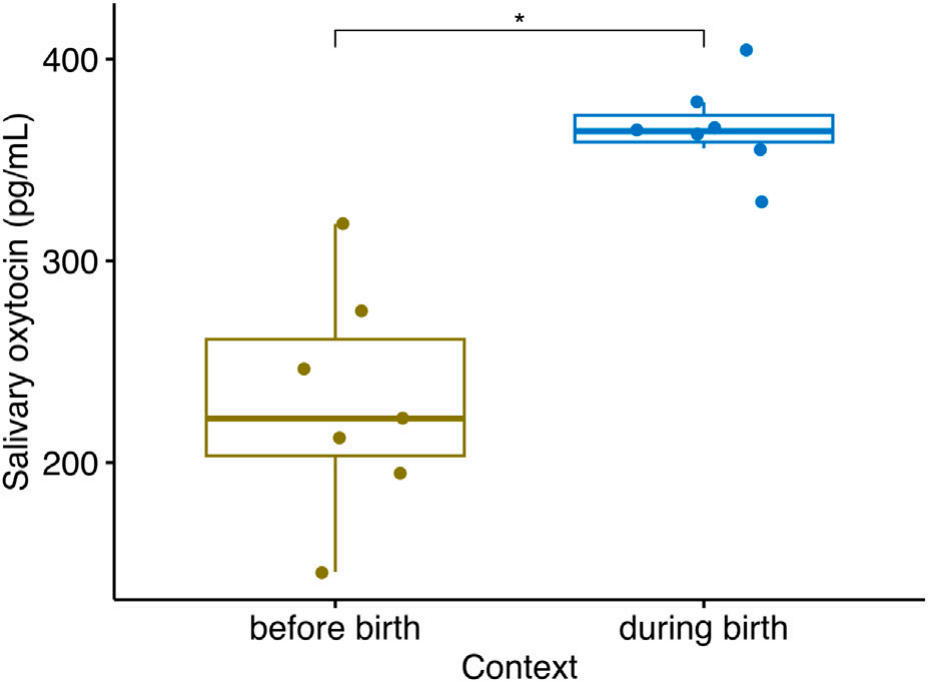
Salivary oxytocin concentrations in *n* = 7 mares sampled between one to 2 days prior to parturition, when they first showed signs of going into labor, and during their parturition (Wilcoxon, V = 0, *n* = 7, *p* = 0.016). Boxplots indicate medians and 25% and 75% interquartile ranges. Error bars represent minimum and maximum values (excluding outliers) *p < 0.05.

Considering extracted samples in mares, sOXT concentrations during the birth were higher than sOXT concentrations in samples collected 1 day prior to birth (Median (25%–75% IQR) = 364 (359–372) pg/mL vs. 222 (203–261) pg/mL, Wilcoxon, *n* = 7, V = 0, *p* = 0.016, see Figure 5).

#### 3.3.2 Salivary oxytocin tends to decrease in sows during a prolonged maternal separation

**FIGURE 6:**
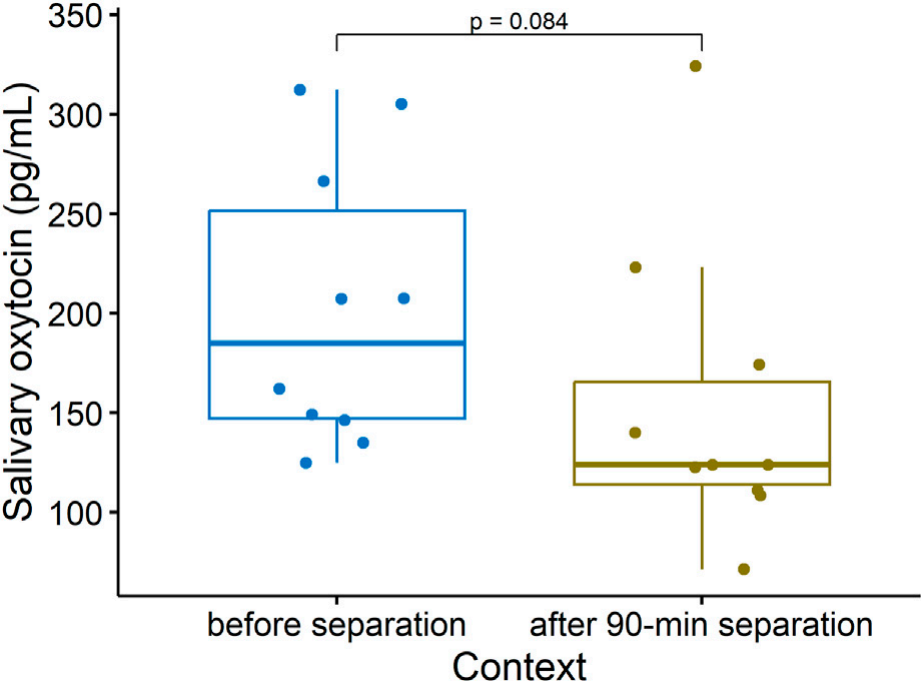
Salivary oxytocin concentrations in lactating sows sampled shortly before being separated from their offspring, and after being separated from their offspring for 90 min (Wilcoxon, v = 45, *n* = 10, *p* = 0.084). Boxplots indicate medians and 25% and 75% interquartile ranges. Error bars represent minimum and maximum values (excluding outliers).

Centrally, oxytocin also plays a critical role in the formation of stable social preferences, or social bonds, the most fundamental of which is the mother-offspring bond (reviewed in Mota-Rojas et al., 2023). However studies have so far failed to relate peripheral OXT concentrations in sows to variation in maternal behavior (Valros et al., 2004) or to 15-minute maternal separations (Hall et al., 2021). We predicted that sOXT would decrease in lactating sows during longer separations from their offspring. Consistent with this hypothesis, sows tended to have decreases in sOXT after being separated from their offspring for 90 min, compared to baseline concentrations collected shortly before the separation (before separation: Median (25%–75% IQR) = 185 (147–252) pg/mL, after 90-min separation: Median (25%–75% IQR) = 124 (114–166) pg/mL, Wilcoxon, *n* = 10, V = 45, *p* = 0.084, see Figure 6).

## 4 Discussion

Given the role of oxytocin, both centrally and peripherally, in mediating salient social processes, the potential to measure peripheral oxytocin non-invasively has important implications, ranging from advancing research into the biological basis of human social disorders (Tabak et al., 2023) to developing biomarkers of positive emotions and welfare in farm animals (Rault et al., 2017). Considering the fast pace of oxytocin research, it is imperative for studies to follow established guidelines (e.g., Andreasson et al., 2015) for reporting full validations of novel in-house assays, and partial validations when using commercially available assays to measure oxytocin in a new species and/or matrix. Many commercially available oxytocin EIA kits are not validated for saliva, and exhibit differences in performance and specificity for sOXT measurement (e.g., dogs: MacLean et al., 2018; humans: Martins et al., 2020). Even when considering the same matrix, samples from different species may exhibit differences in matrix effects which can influence the need for extractions, as well as the optimal extraction procedure to achieve acceptable recovery (Bienboire-Frosini et al., 2017; MacLean et al., 2019; Lürzel et al., 2020). Thus careful consideration of EIA kits and optimization of the extraction method are recommended when validating peripheral oxytocin measurement in a new species or matrix. Greater consistency in reporting validations of different methods for sOXT measurement are important for developing gold standards that will lead to wider acceptance of non-invasive methods for measuring peripheral oxytocin. In addition to analytical validations, biological validations help to determine whether the method is suitable for its intended use.

Low concentrations of sOXT in unextracted pooled pig samples interfered with validations. However extracted samples had higher oxytocin concentrations, which may result in part from cleaving of oxytocin from proteins that interfere with its measurement. Extracted pig samples were validated for precision, parallelism and recovery and sOXT concentrations in extracted samples increased in response to a relevant biological event, parturition. Based on these results, we recommend an extraction step prior to measuring pig sOXT using EIAs. In contrast with pigs, we were able to perform analytical validations of sOXT in unextracted as well as extracted samples from horses, although parallelism and recovery were slightly improved with an extraction step. Moreover, concentrations of sOXT measured in unextracted and extracted duplicates of horse samples were highly correlated. We conclude that sOXT in horses can be measured via EIA without an extraction step, although our results also indicate that an extraction will not interfere with the results, and may in fact improve accuracy. We also demonstrate steps to optimize the extraction process that result in reduced volumes of reagents required for washing and elution and improved recovery compared to previous methods for extracting oxytocin in pig saliva (e.g., Lürzel et al., 2020; Moscovice et al., 2022). Such optimization can increase efficiency while reducing potential sources of human error compared to longer extractions with multiple loadings of wash and elution solutions.

Our biological validation adds to several previous studies showing increases in sOXT in pigs during processes related to reproduction and parturition that are known to be mediated by the oxytocinergic system (López-Arjona et al., 2020a; López-Arjona et al., 2020b). We also show similar sOXT increases during parturition in horses, which have not been previously demonstrated. Our time window for sampling during birth is also narrower compared to other studies (e.g., López-Arjona et al., 2020a; Hall et al., 2021), which allows us to pin-point increases in sOXT specifically related to the birth process itself. We found evidence that pig sOXT concentrations fluctuated within short time-frames during the birth process, since only two of three samples collected within 5-minute intervals during birth differed significantly from post-birth samples. Follow-up longitudinal studies that identify sources of within-individual variation in sOXT concentrations at baseline as well as during salient events are critical for optimizing the timing of sampling and the measurement process to best capture changes in peripheral oxytocin related to events of interest.

Our results also indicate limitations in the sensitivity of our current method for detecting changes in sOXT related to salient social events. We would expect short-term separations from offspring to be associated with reductions in salivary oxytocin in lactating sows, due to a lack of opportunities for nursing and other social contact that is likely mediated by the oxytocinergic system. Our results indicate only a tendency for sOXT to decrease after a 1.5-h separation. Although our sample size was small, our results are similar to previous findings with a larger sample size, in which young pigs also showed tendencies towards reductions in sOXT during short separations from their social group (Moscovice et al., 2022). Moreover, we were able to detect significant differences in sOXT related to the birth process with similarly small sample sizes in both pigs and horses. Interestingly, while there is some evidence for increases in sOXT in farm animals related to positive interactions with humans (pigs: Lürzel et al., 2020; horses: Niittynen et al., 2022), no studies to date have reported similar results related to positive social interactions with conspecifics in farm animals. Further research is thus needed to determine whether sOXT can be used as an indicator of valued social partners and social interactions in farm animals.

Saliva has clear advantages in terms of ease of collection and the potential to work with a relatively clean matrix in comparison with plasma (Gröschl, 2009), where a larger proportion of oxytocin is likely to be bound to proteins, making it more difficult to detect (MacLean et al., 2019). However, methods for quantification of oxytocin in saliva are still relatively new compared to plasma measurements (McCullough et al., 2013; Martins et al., 2020). Two important parameters of saliva that are likely to affect measurement of peptides like oxytocin are flow rate, which is influenced by the autonomic nervous system, and pH, which can influence degradation (Bellagambi et al., 2020). There has been little effort so far to incorporate and control for inter- and intra-individual variation in these parameters on sOXT measurement. Several studies have found that the mode of collection, especially considering whether the saliva sample is unstimulated (e.g., passive drool) or stimulated (e.g., by chewing on swabs) can influence flow rate and subsequent concentrations of peptides and proteins (reviewed in Bellagambi et al., 2020). Most animal studies use swabs for saliva collection, making it likely that samples represent stimulated saliva. However, studies often differ in the swab material used (see Table 1), which can also influence the detection of peptides like oxytocin, with polyester swabs showing better recoveries compared to cotton swabs (Bellagambi et al., 2020). Finally, food and drink can alter saliva flow rate and composition and thus also influence oxytocin measurement (MacLean et al., 2018). Therefore, all efforts should be made to avoid giving subjects access to food or water prior to sampling, which is often hard to achieve in practice in animal studies.

The current diversity in type of collection materials, pre-analytical sample processing and immunoassays used for measurement of sOXT contributes to the often orders of magnitude differences in detected concentrations within the same species and context (e.g., sOXT concentrations in sows on their first lactation day ranging from median (range) = 116 (62–335) pg/mL, this study) to 1,119 (718–2,140) pg/mL, López-Arjona et al., 2020a). The lack of standardization makes it difficult to characterize species-typical ranges of sOXT concentrations and to identify changes in sOXT that are likely to signal biologically-meaningful events for individuals. This problem is exacerbated by a general lack of knowledge regarding causes and extent of individual variability in baseline sOXT concentrations in any species (e.g., pigs: Moscovice et al., 2022; humans: Martins et al., 2020; Caicedo Mera et al., 2021). More efforts are needed to characterize baseline sOXT concentrations longitudinally, and to determine how baseline concentrations are influenced by extraneous factors such as age, body weight, subject sex, individual hydration or physical exercise. This assessment is important for determining whether sOXT can serve as a reliable trait marker for general oxytocin system activation in response to some salient social contexts.

The increase in published methods for the measurement of salivary oxytocin in domesticated animals holds promise for the advancement of research into the biological underpinnings of social behavior and the development of non-invasive indicators of species-specific social needs. However further method standardization and refinement, combined with more biological validations, are required in order to determine if and when changes in peripheral oxytocin relate to specific social events and related emotions. These efforts can be advanced by pairing validated measures of salivary oxytocin with additional validated, non-invasive biomarkers including behavior and physiological indicators of arousal such as cortisol and heart rate variability. A more holistic approach can help to identify valued social contexts for farm animals and can contribute new insights into animal welfare science.

## Data Availability

The original contributions presented in the study are included in the article/Supplementary Material, further inquiries can be directed to the corresponding author.

## References

[B1] Andreasson U. Perret-Liaudet A. van Waalwijk van Doorn L. J. C. Blennow K. Chiasserini D. Engelborghs S. (2015). A practical guide to immunoassay method validation. Front. Neurol. 6, 179. 10.3389/fneur.2015.00179 26347708 PMC4541289

[B2] Baciadonna L. Duepjan S. Briefer E. F. Padilla de la Torre M. Nawroth C. (2018). Looking on the bright side of livestock emotions—the potential of their transmission to promote positive welfare. Front. Vet. Sci. 5, 218. 10.3389/fvets.2018.00218 30258847 PMC6143710

[B3] Bakdash J. Z. Marusich L. R. (2017). Repeated measures correlation. Front. Psychol. 8, 456. 10.3389/fpsyg.2017.00456 28439244 PMC5383908

[B4] Bellagambi F. G. Lomonaco T. Salvo P. Vivaldi F. Hangouët M. Ghimenti S. (2020). Saliva sampling: methods and devices. An overview. Trac. - Trends Anal. Chem. 124, 115781. 10.1016/j.trac.2019.115781

[B5] Bienboire-Frosini C. Chabaud C. Cozzi A. Codecasa E. Pageat P. (2017). Validation of a commercially available enzyme immunoassay for the determination of oxytocin in plasma samples from seven domestic animal species. Front. Neurosci. 11, 524. 10.3389/fnins.2017.00524 28983237 PMC5613128

[B6] Borrow A. P. Cameron N. M. (2012). The role of oxytocin in mating and pregnancy. Horm. Behav. 61, 266–276. 10.1016/j.yhbeh.2011.11.001 22107910

[B7] Caicedo Mera J. C. Cárdenas Molano M. A. García López C. C. Acevedo Triana C. Martínez Cotrina J. (2021). Discussions and perspectives regarding oxytocin as a biomarker in human investigations. Heliyon 7, e08289. 10.1016/j.heliyon.2021.e08289 34805562 PMC8581272

[B8] Caldwell H. K. (2017). Oxytocin and vasopressin: powerful regulators of social behavior. Neuroscientist 23, 517–528. 10.1177/1073858417708284 28492104

[B9] Castrén H. Algers B. de Passillé A. M. Rushen J. Uvnäs-Moberg K. (1993). Early milk ejection, prolonged parturition and periparturient oxytocin release in the pig. Anim. Sci. 57, 465–471. 10.1017/s1357729800042806

[B10] Cerón J. J. Contreras-Aguilar M. D. Escribano D. Martínez-Miró S. López-Martínez M. J. Ortín-Bustillo A. (2022). Basics for the potential use of saliva to evaluate stress, inflammation, immune system, and redox homeostasis in pigs. BMC Vet. Res. 18, 81. 10.1186/s12917-022-03176-w 35227252 PMC8883734

[B11] Cordoni G. Comin M. Collarini E. Robino C. Chierto E. Norscia I. (2023). Domestic pigs (*Sus scrofa*) engage in non-random post-conflict affiliation with third parties: cognitive and functional implications. Anim. Cogn. 26, 687–701. 10.1007/s10071-022-01688-4 36344830 PMC9950185

[B12] Donaldson Z. R. Young L. J. (2008). Oxytocin, vasopressin, and the neurogenetics of sociality. Science 322, 900–904. 10.1126/science.1158668 18988842

[B13] Driscoll C. A. Macdonald D. W. O’Brien S. J. (2009). From wild animals to domestic pets, an evolutionary view of domestication. Proc. Natl. Acad. Sci. U. S. A. 106, 9971–9978. 10.1073/pnas.0901586106 19528637 PMC2702791

[B14] Engelmann M. Landgraf R. Wotjak C. T. (2004). The hypothalamic-neurohypophysial system regulates the hypothalamic-pituitary-adrenal axis under stress: an old concept revisited. Front. Neuroendocrinol. 25, 132–149. 10.1016/j.yfrne.2004.09.001 15589266

[B15] Gan H. W. Leeson C. Aitkenhead H. Dattani M. (2023). Inaccuracies in plasma oxytocin extraction and enzyme immunoassay techniques. Compr. Psychoneuroendocrinology 15, 100188. 10.1016/j.cpnec.2023.100188 PMC10285453 37360277

[B16] Gnanadesikan G. E. Hammock E. A. D. Tecot S. R. Carter C. S. MacLean E. L. (2021). Specificity of plasma oxytocin immunoassays: a comparison of commercial assays and sample preparation techniques using oxytocin knockout and wildtype mice. Psychoneuroendocrinology 132, 105368. 10.1016/j.psyneuen.2021.105368 34364024 PMC8487999

[B17] Gnanadesikan G. E. Hammock E. A. D. Tecot S. R. Lewis R. J. Hart R. Carter C. S. (2022). What are oxytocin assays measuring? Epitope mapping, metabolites, and comparisons of wildtype & knockout mouse urine. Psychoneuroendocrinology 143, 105827. 10.1016/j.psyneuen.2022.105827 35714438 PMC9807061

[B18] Gröschl M. (2009). The physiological role of hormones in saliva. BioEssays 31, 843–852. 10.1002/bies.200900013 19554609

[B19] Hall S. A. Farish M. Coe J. Baker E. Camerlink I. Lawrence A. B. (2021). Minimally invasive biomarkers to detect maternal physiological status in sow saliva and milk. Animal 15, 100369. 10.1016/j.animal.2021.100369 34607115

[B20] Haluska G. J. Currie W. B. (1988). Variation in plasma concentrations of oestradiol-17 beta and their relationship to those of progesterone, 13,14-dihydro-15-keto-prostaglandin F-2 alpha and oxytocin across pregnancy and at parturition in pony mares. J. Reprod. Fertil. 84, 635–646. 10.1530/jrf.0.0840635 3199383

[B21] Huffmeijer R. Alink L. R. A. Tops M. Grewen K. M. Light K. C. Bakermans-Kranenburg M. J. (2012). Salivary levels of oxytocin remain elevated for more than two hours after intranasal oxytocin administration. Neuroendocrinol. Lett. 33, 21–25. 22467107

[B22] Kassambara A. (2023). Pipe-friendly framework for basic statistical tests [R package rstatix version 0.7. 2]. Available at: https://cran.r-project.org/package=rstatix (Accessed January 2, 2024).

[B23] Lee V. E. Arnott G. Turner S. P. (2022). Social behavior in farm animals: applying fundamental theory to improve animal welfare. Front. Vet. Sci. 9, 932217. 10.3389/fvets.2022.932217 36032304 PMC9411962

[B24] Leng G. Sabatier N. (2016). Measuring oxytocin and vasopressin: bioassays, immunoassays and random numbers. J. Neuroendocrinol. 28–12413. jne. 10.1111/jne.12413 PMC5096068 27467712

[B25] Lenth R. Singmann H. Love J. Buerkner P. Herve M. (2019). Package “emmeans.”. Available at: https://cran.microsoft.com/snapshot/2018-01-13/web/packages/emmeans/emmeans.pdf (Accessed February 9, 2023).

[B26] López-Arjona M. Escribano D. Mateo S. V. Contreras-Aguilar M. D. Rubio C. P. Tecles F. (2020c). Changes in oxytocin concentrations in saliva of pigs after a transport and during lairage at slaughterhouse. Res. Vet. Sci. 133, 26–30. 10.1016/j.rvsc.2020.08.015 32919235

[B27] López-Arjona M. Mainau E. Navarro E. Contreras-Aguilar M. D. Escribano D. Mateo S. V. (2021a). Oxytocin in bovine saliva: validation of two assays and changes in parturition and at weaning. BMC Vet. Res. 17, 140. 10.1186/s12917-021-02838-5 33794896 PMC8017845

[B28] López-Arjona M. Mateo S. V. Cerón J. J. Martínez-Subiela S. (2021b). Changes in salivary oxytocin after stroking in dogs: validation of two assays for its assessment. Res. Vet. Sci. 136, 527–534. 10.1016/j.rvsc.2021.04.007 33882381

[B29] López-Arjona M. Mateo S. V. Manteca X. Escribano D. Cerón J. J. Martínez-Subiela S. (2020a). Oxytocin in saliva of pigs: an assay for its measurement and changes after farrowing. Domest. Anim. Endocrinol. 70, 106384. 10.1016/j.domaniend.2019.106384 31569032

[B30] López-Arjona M. Padilla L. Roca J. Cerón J. J. Martínez-Subiela S. (2020b). Ejaculate collection influences the salivary oxytocin concentrations in breeding male pigs. Animals 10, 1268–1312. 10.3390/ani10081268 32722376 PMC7460095

[B31] Lunney J. K. Van Goor A. Walker K. E. Hailstock T. Franklin J. Dai C. (2021). Importance of the pig as a human biomedical model. Sci. Transl. Med. 13, 5758. 10.1126/scitranslmed.abd5758 34818055

[B32] Lürzel S. Bückendorf L. Waiblinger S. Rault J. L. (2020). Salivary oxytocin in pigs, cattle, and goats during positive human-animal interactions. Psychoneuroendocrinology 115, 104636. 10.1016/j.psyneuen.2020.104636 32160578

[B33] MacLean E. L. Gesquiere L. R. Gee N. Levy K. Martin W. L. Carter C. S. (2018). Validation of salivary oxytocin and vasopressin as biomarkers in domestic dogs. J. Neurosci. Methods 293, 67–76. 10.1016/j.jneumeth.2017.08.033 28865986

[B34] MacLean E. L. Wilson S. R. Martin W. L. Davis J. M. Nazarloo H. P. Carter C. S. (2019). Challenges for measuring oxytocin: the blind men and the elephant? Psychoneuroendocrinology 107, 225–231. 10.1016/j.psyneuen.2019.05.018 31163380 PMC6634994

[B35] Marsh N. Marsh A. A. Lee M. R. Hurlemann R. (2020). Oxytocin and the neurobiology of prosocial behavior. Neuroscientist 27, 604–619. 10.1177/1073858420960111 32981445 PMC8640275

[B36] Martins D. Gabay A. S. Mehta M. Paloyelis Y. (2020). Salivary and plasmatic oxytocin are not reliable trait markers of the physiology of the oxytocin system in humans. Elife 9, 624566–e62519. 10.7554/ELIFE.62456 PMC7732341 33306025

[B53] Marchant J. N. Rudd A. R. Mendl M. T. Broom D. M. Meredith M. J. Corning S. (2000). Timing and causes of piglet mortality in alternative and conventional farrowing systems. Vet. Rec. 147, 209–214. 10.1136/vr.147.8.209 10994922

[B37] McCullough M. E. Churchland P. S. Mendez A. J. (2013). Problems with measuring peripheral oxytocin: can the data on oxytocin and human behavior be trusted? Neurosci. Biobehav. Rev. 37, 1485–1492. 10.1016/j.neubiorev.2013.04.018 23665533

[B38] Moscovice L. R. Eggert A. Manteuffel C. Rault J. L. (2023). Spontaneous helping in pigs is mediated by helper’s social attention and distress signals of individuals in need. Proc. R. Soc. B Biol. Sci. 290, 20230665. 10.1098/rspb.2023.0665 PMC10394407 37528710

[B39] Moscovice L. R. Gimsa U. Otten W. Eggert A. (2022). Salivary cortisol, but not oxytocin, varies with social challenges in domestic pigs: implications for measuring emotions. Front. Behav. Neurosci. 16, 899397. 10.3389/fnbeh.2022.899397 35677575 PMC9169876

[B40] Mota-Rojas D. Marcet-Rius M. Domínguez-Oliva A. Martínez-Burnes J. Lezama-García K. Hernández-Ávalos I. (2023). The role of oxytocin in domestic animal’s maternal care: parturition, bonding, and lactation. Animals 13, 1207. 10.3390/ani13071207 37048463 PMC10093258

[B41] Nawroth C. Langbein J. Coulon M. Gabor V. Oesterwind S. Benz-Schwarzburg J. (2019). Farm animal cognition-linking behavior, welfare and ethics. Front. Vet. Sci. 6, 24. 10.3389/fvets.2019.00024 30838218 PMC6383588

[B42] Niittynen T. Riihonen V. Moscovice L. R. Koski S. E. (2022). Acute changes in oxytocin predict behavioral responses to foundation training in horses. Appl. Anim. Behav. Sci. 254, 105707. 10.1016/j.applanim.2022.105707

[B43] Nyuyki K. D. Waldherr M. Baeuml S. Neumann I. D. (2011). Yes, I am ready now: differential effects of paced versus unpaced mating on anxiety and central oxytocin release in female rats. PLoS One 6, e23599. 10.1371/journal.pone.0023599 21858181 PMC3156771

[B44] Ogi A. Mariti C. Baragli P. Sergi V. Gazzano A. (2020). Effects of stroking on salivary oxytocin and cortisol in guide dogs: preliminary results. Animals 10, 708. 10.3390/ani10040708 32325673 PMC7222818

[B45] Rault J. L. van den Munkhof M. Buisman-Pijlman F. T. A. (2017). Oxytocin as an indicator of psychological and social well-being in domesticated animals: a critical review. Front. Psychol. 8, 1521. 10.3389/fpsyg.2017.01521 28955264 PMC5601408

[B46] Ross H. E. Freeman S. M. Spiegel L. L. Ren X. Terwilliger E. F. Young L. J. (2009). Variation in oxytocin receptor density in the nucleus accumbens has differential effects on affiliative behaviors in monogamous and polygamous voles. J. Neurosci. 29, 1312–1318. 10.1523/JNEUROSCI.5039-08.2009 19193878 PMC2768419

[B47] Tabak B. A. Leng G. Szeto A. Parker K. J. Verbalis J. G. Ziegler T. E. (2023). Advances in human oxytocin measurement: challenges and proposed solutions. Mol. Psychiatry 28, 127–140. 10.1038/s41380-022-01719-z 35999276 PMC9812775

[B48] Uvnäs-Moberg K. Ekström-Bergström A. Berg M. Buckley S. Pajalic Z. Hadjigeorgiou E. (2019). Maternal plasma levels of oxytocin during physiological childbirth - a systematic review with implications for uterine contractions and central actions of oxytocin. BMC Pregnancy Childbirth 19, 285–317. 10.1186/s12884-019-2365-9 31399062 PMC6688382

[B49] Valros A. Rundgren M. Špinka M. Saloniemi H. Hultén F. Uvnäs-Moberg K. (2004). Oxytocin, prolactin and somatostatin in lactating sows: associations with mobilisation of body resources and maternal behaviour. Livest. Prod. Sci. 85, 3–13. 10.1016/S0301-6226(03)00114-3

[B50] Vivrette S. Kindahl H. Munro C. Roser J. Stabenfeldt G. (2000). Oxytocin release and its relationship to dihydro-15-keto PGF2alpha and arginine vasopressin release during parturition and to suckling in postpartum mares. Reproduction 119, 347–357. 10.1530/jrf.0.1190347 10864848

[B51] Ward S. A. Kirkwood R. N. Plush K. L. (2019). Effects of oxytocin and carbetocin on farrowing performance. Anim. Reprod. Sci. 205, 88–93. 10.1016/j.anireprosci.2019.04.007 31047762

[B52] Wotjak C. T. Ganster J. Kohl G. Holsboer F. Landgraf R. Engelmann M. (1998). Dissociated central and peripheral release of vasopressin, but not oxytocin, in response to repeated swim stress: new insights into the secretory capacities of peptidergic neurons. Neuroscience 85, 1209–1222. 10.1016/S0306-4522(97)00683-0 9681958

