# Supplementary material for: Changes in salivary oxytocin in response to biologically-relevant events in farm animals: method optimization and usefulness as a biomarker
Source: Front Physiol. 2024 Mar 19;15:1370557. doi: 10.3389/fphys.2024.1370557 (PMC10985263; doi:10.3389/fphys.2024.1370557)
Supplement: Supplementary file 2 [file DataSheet1.DOCX]

Supplementary material, Moscovice and colleagues, 2024; “*Changes in salivary oxytocin in response to biologically-relevant events in farm animals: Method optimization and usefulness as a biomarker*.”

**S1. List of materials and equipment used for extraction and measurement of salivary oxytocin as detailed in the manuscript**

**Reagents**

Acetonitrile (ACN), HPLC Grade (ROTH, Karlsruhe, Germany)

Trifluoroacetic acid (TFA, VWR Chemicals, Darmstadt, Germany)

Ultrapure Water

**Extraction solutions**

Ultra-pure water solution containing 0.1% TFA

Priming solution: 99% ACN / 1% TFA 0.1%

Wash solution: 10% ACN / 90% TFA 0.1%

Elution solution: 60% ACN / 40% TFA 0.1%

**Materials for saliva collection**

SalivaBio® Children’s Swabs (Salimetrics, CA, USA)

Wooden dowels (approx. 200 mm x 3 mm, L x Ø)

Polypropylene round bottom collection tubes (dimensions: 105 mm x 16.8 mm, L x Ø, Sarstedt, Nümbrecht, Germany), with insert for centrifuging (Volume: 5 mL, dimensions: 75 mm x 15 mm, L x Ø, Covetrus, ME, USA).

**Materials for extraction and measurement**

Oasis PriME® HLB cartridges (1mL, 30 mg sorbent, Waters, MA, USA)

Glass tubes

1.5 mL Eppendorf tubes

Pipette tips (variable sizes)

Oxytocin ELISA kit (Cayman Chemical, Biomol, Hamburg, Germany)

**Equipment**

Calibrated variable precision micropipettes (10- 1000 μl)

Vortex mixer

Centrifuge with cooling function (to 4° C)

Resprep® SPE Manifold (Restek, Bad Homburg, Germany)

Chemker 300 PTFE Vacuum Pump (Restek, Bad Homburg, Germany)

SpeedVac® Vacuum concentrator (Thermo Fisher Scientific, MA, USA)

Plate shaker with incubator (to maintain 25° C)

SPECTROstar Nano calibrated microtiter plate reader (BMG Labtech, Ortenberg, Germany)
